# Modeling epidemic dynamics using Graph Attention based Spatial Temporal networks

**DOI:** 10.1371/journal.pone.0307159

**Published:** 2024-07-15

**Authors:** Xiaofeng Zhu, Yi Zhang, Haoru Ying, Huanning Chi, Guanqun Sun, Lingxia Zeng

**Affiliations:** 1 School of Public Health, Xi’an Jiaotong University Health Science Center, Xi’an, China; 2 School of Information Engineering, Hangzhou Medical College, Hangzhou, Zhejiang, China; Zhejiang University of Technology, CHINA

## Abstract

The COVID-19 pandemic and influenza outbreaks have underscored the critical need for predictive models that can effectively integrate spatial and temporal dynamics to enable accurate epidemic forecasting. Traditional time-series analysis approaches have fallen short in capturing the intricate interplay between these factors. Recent advancements have witnessed the incorporation of graph neural networks and machine learning techniques to bridge this gap, enhancing predictive accuracy and providing novel insights into disease spread mechanisms. Notable endeavors include leveraging human mobility data, employing transfer learning, and integrating advanced models such as Transformers and Graph Convolutional Networks (GCNs) to improve forecasting performance across diverse geographies for both influenza and COVID-19. However, these models often face challenges related to data quality, model transferability, and potential overfitting, highlighting the necessity for more adaptable and robust approaches. This paper introduces the Graph Attention-based Spatial Temporal (GAST) model, which employs graph attention networks (GATs) to overcome these limitations by providing a nuanced understanding of epidemic dynamics through a sophisticated spatio-temporal analysis framework. Our contributions include the development and validation of the GAST model, demonstrating its superior forecasting capabilities for influenza and COVID-19 spread, with a particular focus on short-term, daily predictions. The model’s application to both influenza and COVID-19 datasets showcases its versatility and potential to inform public health interventions across a range of infectious diseases.

## 1 Introduction

Epidemic prediction has been a long-standing challenge in public health, with significant implications for disease control and prevention. Accurate prediction of disease spread enables policymakers to allocate resources effectively, implement targeted interventions, and communicate risks to the public. The COVID-19 pandemic has further emphasized the critical role of epidemic prediction in managing global health crises. The ability to anticipate the trajectory of an outbreak and its potential impact on healthcare systems and society at large is essential for effective preparedness and response. The COVID-19 pandemic has exposed the limitations of traditional epidemiological models and highlighted the urgent need for advanced predictive models capable of accurately forecasting the trajectory of infectious diseases. Timely and precise epidemic forecasting is crucial for effective public health planning, proactive response, and mitigation of adverse impacts. Advancing predictive modeling techniques is significant not only for guiding public health interventions but also for deepening our understanding of disease dynamics. This understanding contributes to the broader field of epidemiological research and its application in managing current and future health crises. Our study primarily aims to provide accurate and timely forecasts of influenza dynamics at a daily resolution, which is crucial for optimizing public health interventions and resource allocation.

Traditional epidemiological models have heavily relied on time series analysis to forecast the trajectory of diseases, focusing primarily on temporal patterns of infection rates. Kane et al. and Zhu et al. have demonstrated the effectiveness of machine learning and deep learning methods, such as Random Forest, LSTM, and attention mechanisms, in predicting avian influenza and real-time influenza outbreaks, respectively [1, 2]. Other research has integrated time series analysis with machine learning for COVID-19 spread prediction in Greece and employed the Facebook Prophet model for outbreak peak forecasting, achieving significant accuracy [3, 4]. Petropoulos et al. and Chhabra et al. proposed statistical models for short-term COVID-19 forecasts and epidemic trend predictions, respectively, supporting healthcare management and policy implementations with their findings [5, 6]. These advancements underline a shift towards leveraging complex data and sophisticated algorithms for more accurate and timely infectious disease forecasting. However, a common challenge across these studies is their reliance solely on temporal data, overlooking the critical dimension of spatial information in modeling and predicting disease spread.

In light of the limitations of traditional time series models, recent studies have pivoted towards integrating spatial dynamics with machine learning for enhanced epidemic forecasting. This progression underscores the critical need for models that can accurately capture the complex interplay between spatial and temporal factors in disease spread. Graph attention mechanisms, which can effectively capture the complex spatial-temporal dependencies in disease spread, have shown great potential in enhancing prediction accuracy and model interpretability. A research leverage graph neural networks and transfer learning to model COVID-19 spread based on human mobility and regional infection history, significantly enhancing pandemic forecasting across countries. The reliance on accurate mobility data and the assumption of uniform model transferability across diverse epidemiological contexts may limit the model’s applicability in varying global health landscapes [7]. The integration of Transformer and GCN for COVID-19 forecasting represents an innovative approach, enhancing prediction performance [8]. However, its dependency on high-quality, region-specific data might limit the model’s effectiveness, raising concerns about its scalability and adaptability to areas with sparse or inconsistent data reporting. ATMGNN is introduced, a spatio-temporal graph neural network that effectively predicts COVID-19 dynamics by integrating spatial and temporal data, including New Zealand’s case studies. However, the model’s complexity and potential overfitting to specific regional data might limit its adaptability to diverse global contexts [9]. Cola-GNN is developed, a graph neural network utilizing cross-location attention for ILI prediction, showcasing enhanced long-term forecasting accuracy [10]. However, the model’s reliance on convolutional neural network components without leveraging attention mechanisms and its testing on limited datasets may constrain its predictive scope and generalizability.

This paper presents the Graph Attention based Spatial Temporal (GAST) model, a cutting-edge approach that innovatively incorporates graph attention networks (GAT) to address the previously unmet need for attention mechanisms in graph neural networks for epidemic modeling. The GAST model fills a critical methodological gap by integrating GAT with spatial-temporal modeling, enabling the model to dynamically capture the heterogeneous influence between regions over time, leading to more nuanced and precise predictions. By leveraging the power of GAT, GAST can effectively learn the time-varying importance of different spatial units, thus providing a more comprehensive and realistic representation of the complex spatial-temporal dynamics in influenza spread. Furthermore, by focusing on daily rather than weekly forecasts, GAST pushes the boundary of temporal granularity in influenza prediction, providing actionable insights at a finer timescale that can better inform real-time decision-making in epidemic control. The development of GAST represents a significant step forward in the field of influenza forecasting, offering a powerful tool for public health authorities to optimize intervention strategies and resource allocation.

The main contributions of this paper are summarized as follows:

1) Introduction of the GAST model, a novel influenza forecasting framework that innovatively integrates graph attention networks (GAT) with spatial-temporal modeling. GAST leverages GAT to dynamically learn the time-varying importance of spatial units, providing a more nuanced and realistic representation of influenza spread.
2) Extensive experiments on multiple influenza and COVID-19 datasets demonstrate GAST’s superior performance, particularly in daily forecasting. The improved temporal resolution and robustness of GAST validated across diverse datasets highlight its potential for informing real-time epidemic response.

The rest of this article is organized as follows. Section 2 reviews the related works of the method of epidemic prediction, and the description of our proposed GAST is given in Section 3. Next, the comprehensive experiments are conducted in Section 4. Finally, Section 5 makes a conclusion of the whole work.

## 2 Ralated work

### 2.1 Time series prediction for epidemic

Traditional epidemiological models have heavily relied on time series analysis to forecast the trajectory of diseases, focusing primarily on temporal patterns of infection rates. Kane et al. and Zhu et al. have demonstrated the effectiveness of machine learning and deep learning methods, such as Random Forest, LSTM, and attention mechanisms, in predicting avian influenza and real-time influenza outbreaks, respectively [1, 2]. Additionally, LSTM networks have been applied to forecast COVID-19 transmission in Canada and America, with studies highlighting the benefits of social distancing and contact tracing [11, 12]. Liu et al. utilized the Nonlinear Auto-Regressive neural network for COVID-19 trend analysis, comparing its performance with traditional models [13]. Other research has integrated time series analysis with machine learning for COVID-19 spread prediction in Greece and employed the Facebook Prophet model for outbreak peak forecasting, achieving significant accuracy [3, 4]. Petropoulos et al. and Chhabra et al. proposed statistical models for short-term COVID-19 forecasts and epidemic trend predictions, respectively, supporting healthcare management and policy implementations with their findings [5, 6].

These advancements underline a shift towards leveraging complex data and sophisticated algorithms for more accurate and timely infectious disease forecasting. However, a common challenge across these studies is their reliance solely on temporal data, overlooking the critical dimension of spatial information in modeling and predicting disease spread. The reliance on purely temporal data in different regions can lead to incomplete understanding of the disease transmission process. Incorporating spatial information and allowing for heterogeneous spatial-temporal patterns is crucial for developing more comprehensive and reliable epidemic forecasting models. This critical gap in existing methods highlights the need for innovative approaches that can effectively integrate spatial and temporal dimensions, enabling a more accurate representation of the complex nature of the spread of infectious diseases.

### 2.2 Spatio-temporal model for epidemic

Spatio-temporal prediction tasks have widespread applications in various domains, such as traffic forecasting, climate modeling, and disease spread prediction [14, 15]. Graph attention mechanisms have shown great potential in enhancing prediction accuracy and model interpretability by effectively capturing the complex spatial-temporal dependencies in disease spread. Deng et al. introduced Cola-GNN, a graph neural network utilizing cross-location attention for ILI prediction, showcasing enhanced long-term forecasting accuracy [10]. However, the model’s reliance on convolutional neural network components and limited dataset testing may constrain its predictive scope and generalizability. Several studies have focused on developing spatio-temporal models for COVID-19 forecasting. Giuliani et al. utilized a mixed-effects generalized linear model to analyze the effectiveness of containment measures in Italy, while Nikparvar et al. developed a deep LSTM neural network leveraging mobility data for enhanced prediction accuracy in US counties [16, 17]. Ganesan and Subramani introduced a predictive modeling framework utilizing high-dimensional partial differential equations, and Wang et al. developed a machine learning framework that integrates a county-level spatiotemporal epidemiological model to assess the risk of COVID-19 in Germany [18, 19]. These approaches provide valuable insights but may face challenges in capturing complex non-linear relationships, ensuring model transferability, and addressing computational complexity. Efforts to integrate real-time data and advanced deep learning techniques have shown promising results. Da Silva et al. introduced a real-time spatio-temporal analysis tool utilizing machine learning for COVID-19 forecasting in Brazil, while Gao et al. developed STAN, a spatio-temporal attention network that outperforms traditional models in both long-term and short-term forecasts [20, 21]. Li et al. and Yu et al. explored the integration of Transformer and GCN architectures, demonstrating enhanced prediction performance but highlighting the need for high-quality, region-specific data [8, 22]. LSTM-CA model is introduced that integrates spatio-temporal analysis for accurate prediction of COVID-19 transmission, demonstrating superior performance in both spatial and temporal precision during the early stages of the epidemic in China [23]. The model’s ability to capture both local and global spatial dependencies through cellular automata is a notable strength. A transformer-based model named EpiGNN is proposed for predicting the spread of COVID-19. EpiGNN incorporates a graph neural network to capture the spatial dependencies among different regions and employs a transformer encoder to model the temporal evolution of the epidemic [24].

In conclusion, the integration of spatial dynamics and advanced machine learning techniques has significantly advanced epidemic prediction. Despite the progress made by these studies in integrating spatial dynamics and machine learning techniques for epidemic prediction, there are still notable limitations that need to be addressed. Many of these models rely on specific data sources or assumptions that may not be valid across different regions or disease scenarios. Furthermore, the interpretability and computational efficiency of these models often remain challenges, hindering their practical application in real-time decision making. However, challenges remain in ensuring model generalizability, scalability, and adaptability to diverse data sources and geographical contexts. Future research should focus on addressing these limitations to develop more robust and comprehensive epidemic forecasting models that can inform effective public health interventions and policy decisions.

## 3 Methodology

### 3.1 Overview

**Fig 1:**
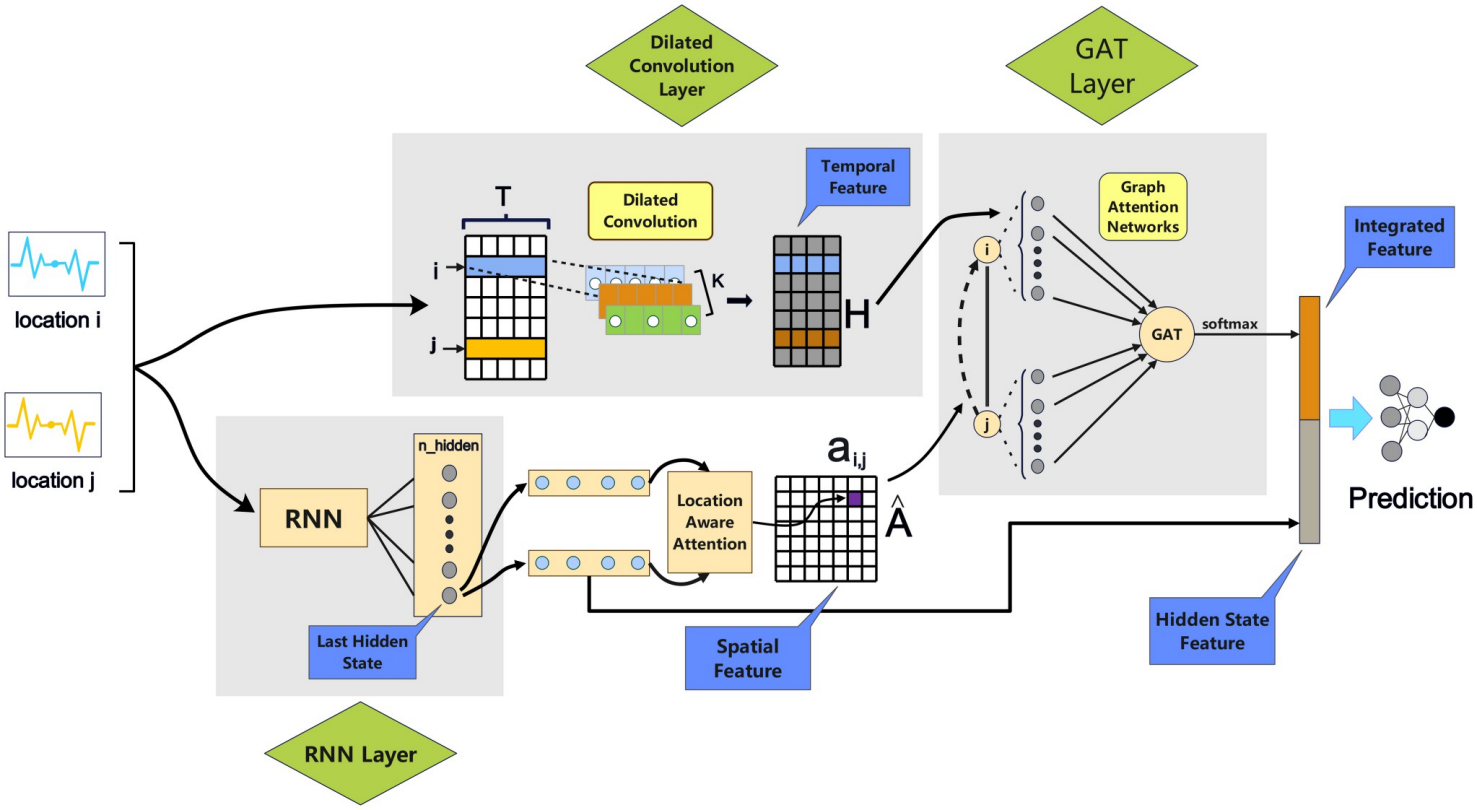
The overall structure diagram of the model. The time series of input data is duplicated into two copies: (1)one passing through the dilated convolution model on the top to learn temporal features; (2)the other passing through the RNN model on the bottom to learn spatial features.

To provide a clear and intuitive understanding of the GAST model architecture, we present a high-level overview in Fig 1. This figure illustrates the key components of the model and their interconnections, serving as a visual guide for readers before delving into the technical details.

As shown in Fig 1, the GAST model consists of three main components: the dilated convolution layer, the RNN layer, and the graph attention layer (GAT). The input data is first duplicated and fed into the Dilated Convolution Layer and the RNN Layer simultaneously. The Dilated Convolution Layer captures multi-scale temporal patterns, while the RNN Layer models spatial dependencies. The outputs of these two layers, referred to as the Temporal Feature and Spatial Feature, respectively, are then fused in the GAT Layer, which leverages self-attention mechanisms to dynamically integrate spatial-temporal information. This design enables GAST to effectively capture the complex interplay of spatial and temporal factors in influenza dynamics, facilitating accurate and timely forecasting. Fig 1 also highlights the flow of information through the model. The input data undergoes feature extraction in the Dilated Convolution Layer and the RNN Layer, and the resulting features are combined in the GAT Layer. The GAT Layer generates an Integrated Feature that incorporates both temporal and spatial information. Finally, the Integrated Feature is concatenated with the Hidden State Feature from the RNN Layer and fed into the output layer for prediction.

The input data is first duplicated into two streams, which are then fed into the Dilated Convolution Layer and the RNN Layer, respectively. This dual-stream architecture allows the model to extract complementary features from both temporal and spatial perspectives.

The Dilated Convolution Layer is employed to capture multi-scale temporal patterns in the influenza incidence series. By applying dilated convolutions with different dilation rates, the model can learn both short-term and long-term temporal dependencies. The output of this layer is referred to as the “Temporal Feature”, which encodes the time-varying dynamics of influenza spread.

Simultaneously, the RNN Layer processes the other stream of input data to capture spatial dependencies and patterns. The RNN is well-suited for modeling sequential data and can effectively learn the spatial context of influenza outbreaks across different regions. The output of the RNN Layer, after being refined by a location-aware attention mechanism, is denoted as the “Spatial Feature”. Additionally, the RNN Layer generates a “Hidden State Feature” that encapsulates the historical information from its own previous states.

The Temporal Feature and Spatial Feature are then fused in the GAT Layer, which leverages self-attention mechanisms to adaptively weight the importance of different spatial units over time. The GAT Layer allows the model to dynamically capture the complex interactions between regions, providing a more nuanced representation of the spatial-temporal influenza dynamics. The output of the GAT Layer is referred to as the “Integrated Feature”, which combines information from both the temporal and spatial domains.

Finally, the Hidden State Feature and the Integrated Feature are concatenated and fed into the output layer for prediction. By incorporating both the temporal context and the spatial dependencies, GAST can generate accurate and fine-grained forecasts of influenza incidence.

The key innovations of GAST lie in its synergistic integration of dilated convolutions, RNN, and graph attention mechanisms, which enables the model to capture the complex spatial-temporal patterns in influenza dynamics at a granular level. The use of graph attention allows GAST to adaptively learn the time-varying importance of different regions, providing a more realistic and interpretable representation of the disease spread process. This powerful combination of techniques empowers GAST to achieve state-of-the-art performance in influenza forecasting, as will be demonstrated in the experiments section.

### 3.2 Temporal convolutional feature extraction

The temporal dynamics of influenza outbreaks exhibit distinct patterns, such as seasonal peaks and troughs, which can provide valuable insights for forecasting future incidence. Convolutional Neural Networks (CNNs) have demonstrated remarkable success in capturing local patterns and dependencies from grid-structured and sequential data [25, 26]. Inspired by this, we introduce a Temporal Convolutional Feature Extraction module to learn multi-scale temporal representations from the input influenza incidence series.

**Fig 2:**
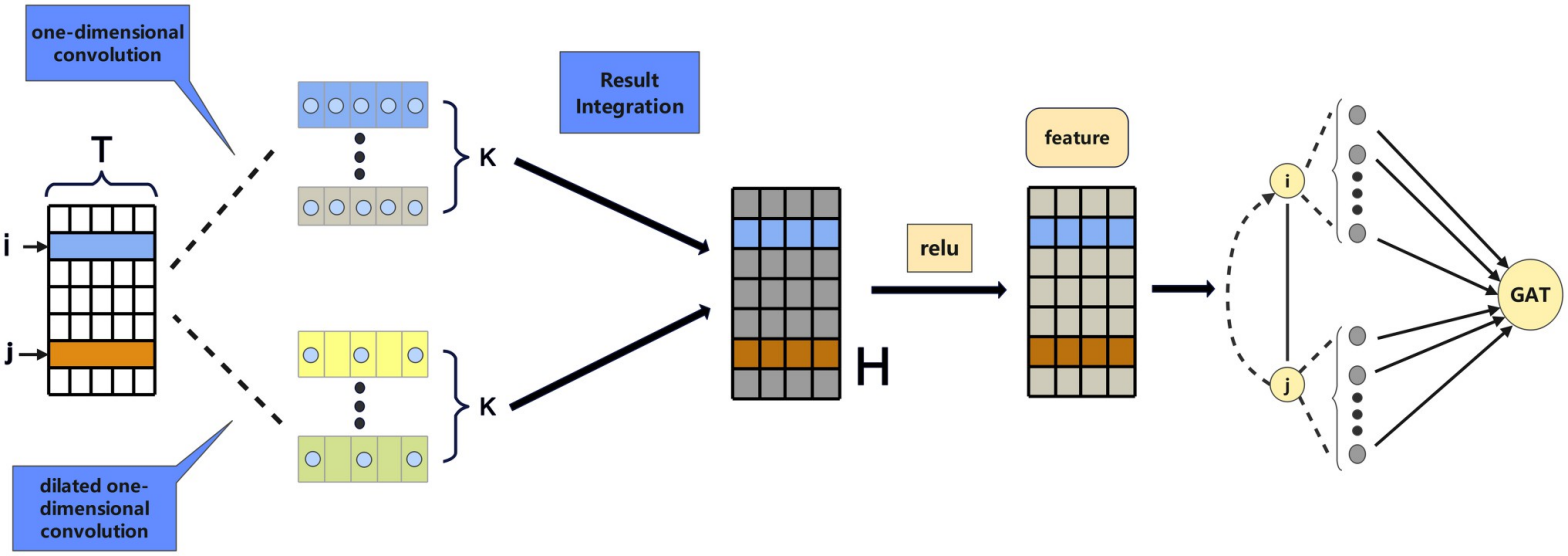
The temporal convolutional feature extraction module consists of two parallel convolutional branches: A standard 1D convolution branch and a dilated 1D convolution branch. The input sequence is processed by both branches simultaneously to capture multi-scale temporal patterns. The outputs from both branches are concatenated to form a feature matrix, which is then passed through a ReLU activation function to generate the final temporal feature matrix.

As illustrated in Fig 2, the module consists of two parallel convolutional branches: a standard one-dimensional convolution branch and a dilated one-dimensional convolution branch. The input sequence is first duplicated and fed into these two branches simultaneously. The outputs from both branches are then integrated and passed to the subsequent GAT module for further processing.

The standard one-dimensional convolution branch applies a set of *k* convolutional filters to the input sequence. Each filter learns to capture specific local patterns or features by convolving a sliding window over the sequence. The convolution operation for the *i*-th element in the output sequence can be formulated as:

$$\begin{array}{c} d_{s}\left[i\right]=\sum_{l=1}^{L}x_{s}\left[i+k\times l\right]\times c\left[l\right] \end{array}$$ (1)

where *x*_*s*_ is the input sequence, *c* is the convolution kernel, *k* is the kernel size, and *L* is the length of the kernel. The kernel size determines the receptive field of each convolution operation, while the number of filters controls the richness of the learned feature representations. By applying multiple filters with different sizes, the model can capture a variety of temporal patterns at different scales.

To further enlarge the receptive field and capture long-range dependencies, we introduce a dilated one-dimensional convolution branch. Dilated convolution is a variant of standard convolution that incorporates a dilation rate *d* to skip over input elements at regular intervals [27]. This effectively increases the receptive field without increasing the number of parameters. The dilated convolution operation can be expressed as:

$$\begin{array}{c} d_{s}\left[i\right]=\sum_{l=1}^{L}x_{s}\left[i+d\times k\times l\right]\times c\left[l\right] \end{array}$$ (2)

By using a larger dilation rate, the dilated convolution branch can capture longer-term temporal patterns and dependencies in the input sequence.

The outputs from both convolutional branches, each consisting of *k* feature maps, are stacked and concatenated along the channel dimension to form a feature matrix **H**. This matrix contains a rich set of multi-scale temporal features learned from the input sequence. To introduce non-linearity and promote feature selectivity, we apply the rectified linear unit (ReLU) activation function to **H**, yielding the final output feature matrix **F**.

The resulting feature matrix **F** is then passed to the subsequent GAT module for spatial dependency modeling and feature integration. By learning both short-term and long-term temporal patterns through the parallel convolutional branches, the Temporal Convolutional Feature Extraction module provides a comprehensive set of temporal features for the downstream forecasting task. The incorporation of dilated convolutions enables the model to capture a wider range of temporal dependencies without significantly increasing the computational complexity. This enhances the model’s ability to learn informative representations from the influenza incidence series, as it can effectively capture both fine-grained and coarse-grained temporal patterns. The multi-scale nature of the learned features allows the model to adapt to the varying temporal dynamics of the disease spread, ultimately contributing to improved forecasting performance.

### 3.3 Directed spatial information extraction

The spread of influenza is not only influenced by temporal factors but also by geographical location. However, previous models often focus solely on extracting information from the time dimension of the input data, neglecting the spatial dimension. To address this issue and fully utilize both temporal and spatial information, our model adopts two different extraction methods: a convolution layer for extracting temporal information, as described previously, and an RNN layer for extracting spatial information, achieving multi-angle information extraction. In this section, we introduce how the model uses the RNN layer to extract spatial information.

Given the RNN model’s strong ability to model sequences and its suitability for capturing spatial dependencies in data, our model employs an RNN by default to extract spatial feature information from the original data. In some cases, the RNN module can be replaced with Gated Recurrent Units (GRU) or Long Short-Term Memory (LSTM) networks. As shown in Fig 1, the prepared original sequence data is fed into the RNN model, with each time step’s input consisting of the feature vector of the geographical location and the number of infections from the previous time step. The RNN layer then calculates the current time step’s hidden state based on the current input and the previous time step’s hidden state. The following equation describes the RNN’s hidden state update process:

$$\begin{array}{c} S_{t}=f\left(U\times X_{t}+W\times S_{t-1}\right) \end{array}$$ (3)

where *S*_*t*_ is the hidden state at time step *t*, *X*_*t*_ is the input vector at time step *t*, and *U* and *W* are the weight matrices for the input and hidden states, respectively. After processing by the RNN layer, the hidden state contains spatial feature information of the geographical location. These hidden states can be regarded as abstract representations of the geographical space, integrating the location’s historical information and current state.

Once the hidden states are calculated, the RNN performs the output computation using the following equation:

$$\begin{array}{c} O_{t}=h\left(V\times S_{t}\right) \end{array}$$ (4)

where *V* is the weight matrix from the hidden state to the output. As depicted in Fig 1, we select the last hidden state for subsequent calculations, as it is considered a summary of the entire sequence information and can provide better generalization ability to the model. The last hidden state, after processing through the geographical adjacency matrix and the attention coefficient matrix (i.e., the “Location-aware attention” in Fig 1), is passed to the subsequent GAT module for further computation.

To enable the model to capture spatial information and historical dependencies in the original sequence and better complete the prediction task, our RNN layer not only calculates the current time step’s hidden state based on the current input and the previous time step’s hidden state but also considers the influence of its own previous time steps’ hidden states. In other words, the model learns the hidden state through its historical sequence. These hidden states are then combined with the output of the GAT network before entering the final prediction stage.

By incorporating the RNN layer for directed spatial information extraction, our model can effectively capture the spatial dependencies and historical context within the input data, leading to a more comprehensive understanding of the influenza spread dynamics and improved prediction performance.

### 3.4 Spatio-temporal Graph Attention Network

In real-world graph structures, the relationships between nodes are often complex and heterogeneous. Traditional Graph Convolutional Networks (GCNs) employ homogeneous neighbor aggregation, assigning equal weights to each neighbor node, which makes it challenging to capture spatial heterogeneity [28]. To address this limitation, we propose a novel approach based on the Graph Attention Network (GAT), a graph neural network model that leverages an adaptive attention mechanism [29]. Compared to traditional GCNs, GAT can assign different attention weights to different nodes, enabling our model to flexibly focus on the diverse relationships between nodes in the graph.

**Fig 3:**
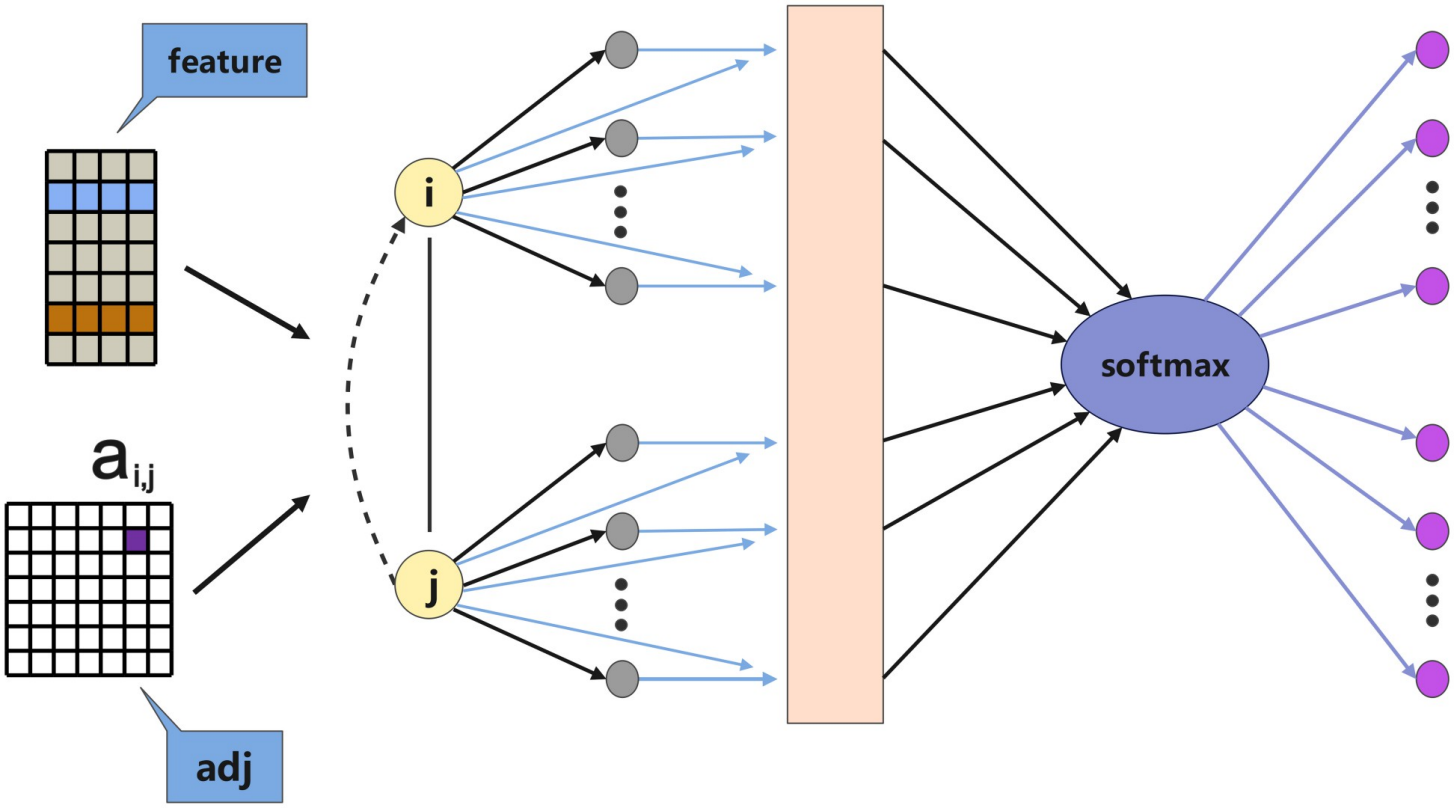
The Graph Attention (GAT) layer takes the temporal feature matrix and the spatial feature matrix as inputs. The GAT layer computes attention scores between nodes using learnable weight matrices. The attention scores are used to weight the importance of neighboring nodes, enabling the model to adaptively capture complex spatial dependencies. The output of the GAT layer is an integrated feature matrix that combines both temporal and spatial information.

As illustrated in Fig 3, the GAT model utilizes a self-attention mechanism to capture the complex relationships between nodes in a graph. To fully exploit this self-attention mechanism, we implement the GAT network as follows. First, we create two learnable two-dimensional weight matrices, denoted as **W** and **a**, where **W** is used for linear transformation of node features, and **a** is used to compute attention scores between nodes. Our model takes the input feature matrix **F** and the graph’s adjacency matrix **A** as inputs. The computations related to these matrices will be detailed in the “Temporal Convolutional Feature Extraction” and “Directed Spatial Information Extraction” sections. We multiply the input feature matrix **F** by the weight matrix **W**, and the result is stored in matrix **H**. Then, we replicate matrix **H** along its second and third dimensions *N* times, where *N* represents the number of features (i.e., the size of the second dimension of matrix **H**). This operation results in matrices **H**_*a*_ and **H**_*b*_ with shapes (*h*_1_, *N* × *h*_2_, *h*_3_) and (*h*_1_, *h*_2_, *N* × *h*_3_), respectively. These two matrices are then concatenated, and the resulting matrix, **A**_*i*_, is used for comparison with other nodes.

Next, we calculate the attention weights by multiplying **A**_*i*_ with the attention weight parameter **a**, obtaining the attention scores between each pair of nodes. The last dimension of the result is compressed to match the shape of matrix **a**, which is (2 × out_features, 1). After applying an activation function, the result is reshaped to (*B*, *N*, *N*), where *B* represents the batch size. This transformation facilitates subsequent calculations of weighted neighbor node representations. The attention mechanism in GAT is completed at this stage, producing the attention weight matrix **E**. We calculate the relationship weights using the following formula:

$$\begin{array}{c} e_{ij}=a\left(\text{W}x_{i},\text{W}x_{j}\right) \end{array}$$ (5)

where *x*_*i*_ and *x*_*j*_ represent the feature vectors of nodes *i* and *j*, respectively. This formula allows us to effectively calculate the strength of relationships between nodes.

To process the attention weight matrix **E**, we create a tensor of the same shape as **E**, which is zeroed out for non-existent edges and denoted as **Z**. We then keep the positions in matrix **E** corresponding to edges present in the adjacency matrix **A**, replacing other positions with the corresponding elements from **Z**. Thus, for non-existent edges, their corresponding attention is set to negative infinity, making them zero in subsequent softmax operations.

To ensure that the sum of attention weights equals 1 for subsequent steps in computing weighted neighbor node representations and to prevent overfitting during model training, we perform a softmax operation on the processed attention weight matrix, obtaining normalized attention coefficients:

$$\begin{array}{c} \alpha_{ij}=\frac{\text{exp}(e_{ij})}{\sum_{k\in N_{i}}\text{exp}\left(e_{ik}\right)} \end{array}$$ (6)

where *α*_*ij*_ measures the importance of node *i* to node *j*, and $N_{i}$ represents the neighborhood of node *i*. This calculation enables our model to focus more on important neighbor nodes, thereby improving the quality and expressiveness of the graph neural network’s node representations. Finally, the attention weight matrix (shape (*B*, *N*, *N*)) is multiplied by the node feature matrix **H** (shape (*B*, *N*, out_features)), constituting the output of the GAT layer:

$$\begin{array}{c} {\text{h}}_{i}^{\left(l+1\right)}=\sigma (\sum_{j\in N_{i}}\alpha_{ij}{\text{W}}^{\left(l\right)}{\text{h}}_{j}^{\left(l\right)}) \end{array}$$ (7)

where **h**_*i*_ represents the hidden representation of node *i* at a certain layer, and **W** is the weight matrix. This step implements the weighted aggregation of neighbor nodes using attention coefficients in GAT.

Overall, the GAT network plays a crucial role in information integration within our model, weighting and aggregating neighbor node information based on attention weights between nodes, thereby obtaining more representative node representations. This approach better captures both local and global information in the graph data structure, effectively improving the accuracy of subsequent prediction tasks.

## 4 Experiment and results

To evaluate the performance of GAST, we conducted a comprehensive comparative study against state-of-the-art forecasting models using diverse datasets from Japan, the United States, and Spain, covering both influenza and COVID-19. Our experimental workflow included data preprocessing, hyperparameter optimization, and model training across different regions to assess robustness and generalizability. We employed Root Mean Square Error (RMSE) and Pearson Correlation Coefficient (PCC) as evaluation metrics to compare the models’ predictive capabilities. Extensive experiments demonstrated that GAST consistently outperformed the compared models, achieving higher accuracy across different datasets and prediction horizons. The successful application of GAST to both influenza and COVID-19 datasets highlights its versatility and adaptability to various infectious disease scenarios, demonstrating its potential for broad application in epidemic forecasting.

### 4.1 Datasets

To comprehensively evaluate the performance and generalizability of the proposed GAST model, we conducted experiments on four real-world epidemic-related datasets, spanning different geographical regions, time periods, and disease types. These datasets include three seasonal influenza datasets from Japan (https://tinyurl.com/y5dt7stm) (prefectural level), the United States (https://tinyurl.com/y39tog3h) (state and regional levels), and a daily COVID-19 dataset from Spain (https://dataforgood.fb.com/tools/disease-prevention-maps/). Table 1 provides a summary of the key characteristics and statistics of each dataset.

**Table 1:** Overview of the datasets used in the experiments.

Dataset	Size	Min	Max	Mean	Granularity
Japan-Prefecture	348 × 47	0	26,635	655	Weekly
US-Region	785 × 10	0	16,526	1,009	Weekly
US-State	360 × 49	0	9,716	223	Weekly
Spain-COVID	122 × 35	0	4,623	38	Daily

“Granularity” indicates the temporal resolution of the epidemic data, while “Size” represents the product of the number of locations and the number of time steps.

The Japan-Prefecture dataset is derived from the Infectious Disease Weekly Report (IDWR) published by the Japanese government. It contains weekly statistics of Influenza-Like Illness (ILI) cases from August 2012 to March 2019 across 47 prefectures in Japan. This dataset allows us to assess the model’s ability to capture the spatiotemporal dynamics of influenza transmission at a fine-grained administrative level.

The US-Region dataset is extracted from the ILINet system, a surveillance network maintained by the United States Health and Human Services (US-HHS). It includes weekly influenza activity levels in ten Health and Human Services (HHS) regions across the continental United States from 2002 to 2017. This dataset enables the evaluation of the model’s performance in capturing regional variations in influenza transmission patterns.

The US-State dataset is obtained from the Centers for Disease Control and Prevention (CDC) and consists of weekly numbers of influenza-like illness visits to healthcare providers from 2010 to 2017 for each state in the USA. Due to incomplete data for one state, we retained data for the remaining 49 states. This dataset allows us to investigate the model’s capability in predicting influenza dynamics at the state level.

The Spain-COVID dataset encompasses daily COVID-19 case data from February 20, 2020, to June 20, 2020, for 35 administrative NUTS3 regions in Spain that were significantly affected by the pandemic. Additionally, this dataset includes human mobility data from Spain provided by the Data for Good program, enabling us to explore the relationship between human mobility and disease transmission. The inclusion of a COVID-19 dataset alongside influenza datasets demonstrates the versatility of the GAST model in adapting to different epidemic scenarios.

The diverse nature of these datasets, spanning different geographical scales, time periods, and disease types, allows for a comprehensive evaluation of the GAST model’s performance and generalizability. By testing the model on multiple real-world scenarios, we can assess its ability to capture complex spatiotemporal patterns, adapt to different data characteristics, and provide accurate predictions for informed decision-making in epidemic response and management.

### 4.2 Evaluation metrics

To comprehensively assess the performance of the GAST model and compare it with existing baselines, we employed two widely used evaluation metrics: Root Mean Square Error (RMSE) and Pearson Correlation Coefficient (PCC). These metrics provide complementary insights into the model’s predictive accuracy and its ability to capture the temporal dynamics of the epidemic data.

RMSE is a standard metric for measuring the average magnitude of the prediction errors. It quantifies the root mean square difference between the predicted values and the corresponding ground truth observations. Mathematically, RMSE is defined as:

$$\begin{array}{c} \text{RMSE}=\sqrt{\frac{1}{n}\sum_{i=1}^{n}{\left(y_{i}-{\hat{y}}_{i}\right)}^{2}} \end{array}$$ (8)

where *y*_*i*_ and ${\hat{y}}_{i}$ denote the actual and predicted values of the epidemic data (e.g., influenza or COVID-19 cases) for the *i*-th data point, respectively, and *n* is the total number of samples. The RMSE is calculated by first computing the squared differences between the predicted and actual values, then taking the average of these squared differences, and finally taking the square root of the average. By squaring the errors, RMSE eliminates the influence of positive and negative errors canceling each other out. A lower RMSE value indicates better predictive accuracy, as it suggests that the model’s predictions are closer to the observed values on average.

While RMSE measures the magnitude of the prediction errors, it does not provide information about the correlation between the predicted and actual values. To capture this aspect, we utilize the Pearson Correlation Coefficient (PCC). PCC is a measure of the linear relationship between two variables, ranging from -1 to +1. A PCC value of +1 indicates a perfect positive linear relationship, -1 indicates a perfect negative linear relationship, and 0 indicates no linear relationship. PCC is defined as:

$$\begin{array}{c} \text{PCC}=\frac{\sum_{i=1}^{n}\left({\hat{y}}_{i}-\bar{\hat{y}}\right)\left(y_{i}-\bar{y}\right)}{\sqrt{\sum_{i=1}^{n}{\left({\hat{y}}_{i}-\bar{\hat{y}}\right)}^{2}}\sqrt{\sum_{i=1}^{n}{\left(y_{i}-\bar{y}\right)}^{2}}} \end{array}$$ (9)

where $\bar{\hat{y}}$ and $\bar{y}$ represent the mean values of the predicted and actual data points, respectively. The numerator of the PCC formula calculates the covariance between the predicted and actual values, which measures the extent to which the two variables vary together. The denominator normalizes the covariance by the product of the standard deviations of the predicted and actual values, ensuring that the PCC is scale-invariant. A higher PCC value indicates a stronger positive linear relationship between the model’s predictions and the ground truth, suggesting that the model effectively captures the temporal trends and patterns in the epidemic data.

By considering both RMSE and PCC, we obtain a comprehensive evaluation of the GAST model’s performance. RMSE assesses the model’s ability to minimize the overall prediction errors, while PCC measures the model’s capability to capture the correlation and temporal dynamics of the epidemic data. Together, these metrics provide a balanced assessment of the model’s predictive accuracy and its alignment with the observed trends.

In the experimental results section, we report the RMSE and PCC values for the GAST model and the compared baselines across different datasets and prediction horizons. We also perform statistical significance tests to validate the superiority of the GAST model over the baselines. By demonstrating significant improvements in both RMSE and PCC, we establish the effectiveness of the GAST model in accurately forecasting epidemic dynamics and its potential for real-world applications in public health decision-making.

### 4.3 Experimental setup

All experiments were conducted on two NVIDIA graphics processing units (GPUs): a GeForce RTX 3090 with 24GB of memory and a GeForce RTX 2080 Ti with 22GB of memory. The programming environment consisted of Python 3.7.4, PyTorch 1.0.1, and CUDA 9.2, ensuring consistent and reproducible results across different runs.

To optimize the performance of the GAST model, we conducted a comprehensive hyperparameter tuning process. The Adam optimizer was used for model training, with the number of epochs set to 1,500 to ensure convergence [30]. We employed a weight decay of 5e-4 as a regularization technique to mitigate overfitting. Additionally, early stopping was implemented based on the validation loss, with a patience of 50 epochs, to further prevent overfitting and reduce computational overhead.

The batch size was set to 32, considering the memory constraints of the GPUs and the efficiency of the training process. An input window size of 20 time steps was used to capture both short-term and long-term dependencies in the epidemic data. The learning rate was fixed at 0.1% throughout the training process, providing a balance between convergence speed and stability.

For the recurrent neural network (RNN) component of the GAST model, we experimented with different numbers of layers and found that setting *nlayer* to 2 yielded the best performance. This configuration allows the model to capture complex temporal patterns while maintaining computational efficiency.

In the multi-scale dilated convolution module, the number of filters *k* was fixed at 10, balancing model complexity and performance. The short-term dilation rate *ks* and long-term dilation rate *kl* were set to 1 and 2, respectively, enabling the model to capture both fine-grained and coarse-grained temporal patterns in the epidemic data.

To evaluate the model’s performance under different forecasting scenarios, we conducted experiments with varying prediction lengths for the influenza and COVID-19 prediction tasks. For the influenza prediction task, we used prediction lengths of 2, 3, 5, 10, 15 weeks, aligned with the weekly granularity of the influenza datasets. For the COVID-19 prediction task, we used prediction lengths of 3, 5, 7, 11, 15 days, corresponding to the daily granularity of the COVID-19 dataset. These different prediction lengths allow us to assess the model’s ability to generate both short-term and long-term forecasts, providing insights into its performance under various public health decision-making scenarios.

### 4.4 Results

We compare our model with several advanced methods in the forecasting domain listed below, along with their variants. These methods encompass a wide range of techniques, from classical time series models to deep learning-based approaches, providing a comprehensive benchmark for evaluating the effectiveness of our model.

GAR, The innovative GAR model, constructed through GM and AR models, was utilized for pioneering prediction experiments on rice blast in Linchuan County from 1988 to 1992. The forecasting accuracy was evaluated using FUZZY functions, revealing the model’s effectiveness in predicting rice blast.

AR [31], The combination of Autoregressive models (AR) with compartment models was employed in innovative experiments, developing a new model for accurately predicting long-term blood glucose levels, thus optimizing diabetes treatment.

VAR [32], The VAR model was used to analyze the China energy-economy-environment system, forecasting up to 2015 based on data from 1995 to 2006. Results indicated that this model could reliably explore the dynamics among the three aspects.

ARMA [33], The ARMA model was utilized for time series analysis of the traditional Chinese patent medicine industry’s output during the “Twelfth Five-Year Plan” period in China, innovatively establishing a forecasting model that revealed the industry’s development trends and patterns during a specific period.

RNN [34], Basic backpropagation methods and Backpropagation Through Time (BPTT) techniques were used for innovative experiments on Recurrent Neural Networks (RNN), establishing models for applications such as pattern recognition. In-depth analysis revealed RNN’s particular suitability for sequence data, demonstrating its great potential and benefits in time series analysis and fault diagnosis.

RNN+Attn [35], Long Short-Term Memory networks (LSTM) were improved by integrating memory networks and attention mechanisms (attn), forming a novel RNN model capable of handling structured inputs. This model demonstrated superior performance in tasks such as language modeling, sentiment analysis, and natural language inference through experiments.

DCRNN [36], The Diffusion Convolutional Recurrent Neural Network (DCRNN) was proposed for traffic flow prediction. This model, simulating diffusion processes on directed graphs and employing an encoder-decoder architecture to capture spatial and temporal dependencies, showed an average prediction accuracy improvement of 12%-15% over existing models on real traffic data.

CNNRNN-Res [37], The CNNRNN-Res framework, combining RNN and CNN with the introduction of a residual structure, aimed to predict epidemiological time series data, reducing the issue of overfitting. This model effectively integrated multiple data sources, surpassing traditional autoregressive models in prediction performance on datasets from the United States and Japan.

LSTNet [38], A deep learning framework named Long- and Short-term Time-series network (LSTNet) was proposed, combining CNN and RNN to capture both long- and short-term patterns in multivariate time series, optimizing performance through conventional autoregressive models. On real complex datasets, LSTNet showed significant performance improvements over other advanced methods.

ST-GCN [39], The Spatio-Temporal Graph Convolutional Network (STGCN) method was innovatively used to tackle the time series prediction challenge of urban traffic flow, constructing a fully convolutional structure model on graphs for faster training speeds and fewer parameters. By modeling multi-scale traffic networks, STGCN effectively captured comprehensive spatio-temporal correlations.

Cola-GNN [40], Innovative experiments using the Cola-GNN model for long-term forecasting of influenza-like illnesses demonstrated the model’s strong predictive capability and significant advantages in handling spatio-temporal dependencies.

We evaluated each model mentioned above under both short-term and long-term forecast weeks settings. The experimental results and comparative analyses for three groups of influenza datasets and two groups of COVID-19 datasets are presented in Tables 2 and 3, respectively. The general trend is that as the level of forecasting increases, the problem becomes more challenging, leading to a decrease in forecasting accuracy. The RMSE differences between datasets are significant, due to the varying scales and variances of the datasets.

**Table 2:** RMSE and PCC metrics for different models on the first three datasets with forecast weeks set at 2, 3, 5, 10, 15.

	Japan-Prefectures					US-Regions					US-States				
RMSE(↓)	2	3	5	10	15	2	3	5	10	15	2	3	5	10	15
GAR	1235	1625	1988	2110	2039	535	715	993	1369	1466	149	187	236	312	354
AR	1377	1705	2012	2111	2043	568	754	1000	1333	1403	162	202	252	308	327
VAR	1341	1714	2031	1935	1897	732	860	1055	1276	1312	241	258	275	306	335
ARMA	1370	1701	2013	2108	2042	561	739	986	1334	1393	162	198	248	308	327
RNN	1016	1242	1197	1622	1633	494	**651**	**880**	1271	1445	143	187	**213**	267	283
RNN+Attn	1116	1362	1781	1598	1966	505	696	984	1295	1290	151	450	231	307	347
DCRNN	1454	1736	2013	2114	2035	741	890	1151	1427	1514	163	195	242	290	293
CNNRNN-Res	1234	1643	1977	1795	1762	558	724	978	1168	1310	239	282	276	**239**	246
LSTNet	1304	1654	2000	1874	1887	533	847	995	1186	**1231**	228	253	285	293	286
ST-GCN	997	**1086**	**1114**	1551	**1311**	718	815	1067	**1156**	1401	190	209	248	280	299
Cola-GNN	1080	1182	1130	1467	1518	**485**	667	924	1270	1367	161	178	229	263	**233**
GAST	**970**	1110	1234	**1441**	1474	488	675	939	1338	1294	**140**	**175**	294	257	244
%relative gain	2.7%	-	-	1.8%	-	-	-	-	-	-	2.1%	1.7%	-	-	-
PCC(↑)	2	3	5	10	15	2	3	5	10	15	2	3	5	10	15
GAR	0.803	0.626	0.339	0.220	0.517	0.932	0.881	0.792	0.581	0.483	0.944	0.914	0.875	0.780	0.712
AR	0.752	0.579	0.309	0.208	0.484	0.928	0.878	0.792	0.622	0.526	0.939	0.909	0.863	0.771	0.720
VAR	0.762	0.581	0.299	0.436	0.464	0.864	0.809	0.686	0.514	0.449	0.845	0.820	0.800	0.742	0.706
ARMA	0.754	0.580	0.310	0.239	0.489	0.927	0.878	0.796	0.604	0.514	0.938	0.910	0.862	0.770	0.721
RNN	0.887	0.844	0.859	0.699	0.710	0.941	0.898	**0.821**	**0.664**	0.497	0.948	0.920	0.885	0.833	0.807
RNN+Attn	0.853	0.784	0.570	0.740	0.333	0.940	0.897	0.798	0.565	0.536	0.948	**0.985**	**0.889**	0.795	0.724
DCRNN	0.725	0.566	0.312	0.224	0.556	0.902	0.858	0.774	0.653	0.628	0.945	0.924	**0.889**	0.838	0.836
CNNRNN-Res	0.821	0.624	0.336	0.599	0.578	0.922	0.863	0.753	0.585	0.435	0.878	0.816	0.814	**0.843**	0.856
LSTNet	0.775	0.604	0.334	0.443	0.484	0.941	0.850	0.734	0.567	0.557	0.891	0.853	0.805	0.778	0.810
ST-GCN	0.901	0.883	0.873	0.744	**0.822**	0.874	0.850	0.744	0.649	**0.722**	0.907	0.888	0.835	0.793	0.767
Cola-GNN	0.890	0.869	**0.888**	0.796	0.759	**0.944**	**0.904**	0.819	0.644	0.569	0.943	0.924	0.874	0.810	0.863
GAST	**0.917**	**0.899**	0.881	**0.806**	0.776	0.943	0.903	0.816	0.558	0.494	**0.956**	0.933	0.767	0.838	**0.867**
%relative gain	1.8%	1.8%	-	1.3%	-	-	-	-	-	-	0.8%	-	-	-	0.4%

Bold indicates the best result in each column, underline indicates the second-best result. Relative gain is compared with the second-best result. The table presents the performance of various models across three datasets (Japan-Prefectures, US-Regions, and US-States) for different forecast horizons. The evaluation metrics used are Root Mean Square Error (RMSE) and Pearson Correlation Coefficient (PCC). Lower RMSE values and higher PCC values indicate better performance. The proposed GAST model demonstrates competitive results, achieving the best or second-best performance in several cases, with relative gains compared to the second-best model.

**Table 3:** RMSE metrics for different models on the last dataset with forecast weeks set at 3, 5, 7, 11, 15.

	Spain-COVID				
RMSE (↓)	3	5	7	11	15
GAR	169	197	203	221	231
AR	216	253	255	226	221
VAR	227	223	216	185	204
ARMA	201	237	243	474	564
DCRNN	159	196	199	217	847
CNNRNN-Res	212	220	237	226	225
LSTNet	**160**	195	214	198	217
Cola-GNN	163	182	177	**171**	196
GAST	177	**168**	**165**	173	**195**
%relative gain	-	7.7%	6.8%	-	0.5%

The table compares the performance of various models on the Spain-COVID dataset using RMSE metric. Bold indicates the best result, underline the second-best, and relative gain compares GAST with the second-best model. GAST achieves competitive performance across different forecast horizons.

In Table 2, the vertical comparison of forecasting accuracy (RMSE) and correlation (PCC) between forecasted and actual values for all mentioned models across three data groups for forecast lengths of 2, 3, 5, 10, 15 weeks, demonstrates our model’s excellent performance on both evaluation metrics. For the ‘Japan-Prefectures’ and ‘US-States’ data groups, our model generally achieved the lowest RMSE values. Overall, our model had the best RMSE values in 4 outcomes: ‘Japan-Prefectures’ at 2 and 10 weeks, ‘US-States’ at 2 and 3 weeks, outperforming the best results of the remaining models by 2.7%, 1.8%, 2.1%, and 1.7% in forecasting accuracy, respectively. Moreover, in 5 out of 15 RMSE values, our model achieved the second-best results. In terms of PCC, our model also showed strong performance, maintaining high scores in different steps for ‘Japan-Prefectures’, ‘US-Regions’, and ‘US-States’, indicating that our model’s forecasted trends are highly consistent with actual values. The model achieved the best PCC values in 5 outcomes: ‘Japan-Prefectures’ at 2, 3, and 10 weeks, ‘US-States’ at 2 and 15 weeks, surpassing the best results of other models by 1.8%, 1.8%, 1.3%, 0.8%, and 0.4% in accuracy, respectively, with six other datasets achieving the second-best among all models. Although our model was not always the best in all indicators and forecast periods, its results were generally within the range of better performance. Compared to other models, the predictive performance improvements brought by our model were significant in some cases, demonstrating its versatility and stability.

In Table 3, comparing our model’s forecasting accuracy (RMSE) with that of the mentioned models for COVID-19 forecasting tasks at forecast lengths of 3, 5, 7, 11, 15 days, our model also performed well in COVID-19 forecasting tasks. During COVID-19, due to governmental interventions (such as stay-at-home orders, lockdowns), there were significant differences in epidemic situations across regions, so we only selected data from Spain. Unlike the other three data groups, the forecast lengths for the Spanish data group were measured in days. We found our model achieved the best forecasting accuracy for forecast lengths of 5, 7, 15 days, surpassing the optimal forecasting accuracy of the remaining models by 7.7%, 6.8%, 0.5%, respectively. Our model is competitive in forecasting COVID-19, which has transmission mechanisms similar to, yet distinct from, influenza. This proves that our model can be further applied in a broader range of fields and perform well in forecasting tasks.

To investigate the factors contributing to the performance differences among the models, we conducted an analysis focusing on three key aspects:

- **Spatial-Temporal Dependency Capture**: Models that effectively integrate both spatial and temporal information generally outperform those relying solely on temporal data. GAST’s superior performance stems from its unique combination of graph attention networks (GAT) and dilated convolutions, enabling it to adaptively capture complex spatial dependencies and learn multi-scale temporal patterns.
- **Model Architecture and Design**: The performance differences can be traced back to the models’ architectural designs. Traditional time-series models lack the ability to capture spatial dependencies and nonlinear relationships, while deep learning-based models vary in their effectiveness depending on how well they integrate spatial and temporal information. GAST’s architecture, combining graph attention, dilated convolutions, and a location-aware attention mechanism, enables it to model the spatial-temporal dynamics more accurately than other models.
- **Robustness and Versatility**: GAST’s consistent performance across different datasets, including both influenza and COVID-19, demonstrates its robustness and versatility. The model’s ability to adapt to different geographical scales, disease dynamics, and forecast horizons highlights its potential for broad application in various epidemiological scenarios.

In summary, GAST’s superior performance in forecasting COVID-19 and influenza can be attributed to its effective integration of spatial-temporal modeling techniques, advanced architecture design, and robustness and versatility across different datasets and scenarios.

### 4.5 Abalation study

In Table 4, we conducted ablation experiments on three groups of influenza datasets: Japan-Prefectures, US-Regions, and US-States, selecting forecast lengths of 2 weeks and 10 weeks for short-term and long-term predictions, respectively, to better compare and analyze the performance of the GAT graph structure in different forecasting tasks. We set the graph structure in our model to GAT, GCN, and no graph structure, with the performance metric being RMSE, to compare their performances. On the Japan-Prefectures dataset, the GAT structure improved by 10.2% over GCN for the 2-week prediction and also saw a slight increase of 1.8% for the 10-week prediction. In the US-Regions dataset, GAT’s performance was slightly lower than GCN for the 2-week prediction but did poorly for the long-term prediction of 10 weeks. On the other hand, in the US-States dataset, the GAT structure achieved good results for both short-term and long-term predictions, especially achieving a 13% relative improvement in the short-term and a 2.3% improvement in the long-term. Additionally, the no graph structure also performed well in specific cases, such as achieving the best RMSE score for the 10-week prediction in the US-Regions dataset. Overall, the GAT graph structure used in our model is more suited to solving this type of influenza prediction problem compared to GCN and no graph structure. Especially for long-term predictions, GAT demonstrated its potential in handling predictions over larger time spans. The performance of each model on three datasets of varying complexity reveals their applicability in different environments and contexts, indicating that GAT can provide more accurate results for influenza prediction in most cases.

**Table 4:** Ablation experiments on graph structures and RMSE metrics for the first three groups of influenza datasets with forecast weeks at 2 and 10.

RMSE(↓)						
Graph Structure	JP-Pref		US-Reg		US-Sta	
	2	10	2	10	2	10
Baseline	1049	1579	522	**1269**	**140**	278
Baseline + GCN	1080	1467	**485**	1270	161	263
**Baseline + GAT (ours)**	**970**	**1441**	488	1338	**140**	**257**
%relative gain	10.2%	1.8%	-	-	13%	2.3%

The table presents ablation experiment results on different graph structures for three influenza datasets. Bold indicates the best result, underline indicates the second-best, and relative gain compares our model (Baseline + GAT) with the second-best. The results demonstrate the effectiveness of incorporating GAT in our model.

The ablation experiments provide valuable insights into the effectiveness of different graph structures for influenza prediction tasks. The results demonstrate the superiority of the GAT structure in capturing the complex spatial dependencies present in the influenza data. The attention mechanism employed by GAT allows the model to adaptively assign different weights to neighboring nodes, enabling it to focus on the most relevant spatial relationships. This is particularly evident in the Japan-Prefectures and US-States datasets, where GAT consistently outperforms GCN and no graph structure in both short-term and long-term predictions. Furthermore, the ablation study highlights the robustness of the GAT structure across different prediction lengths, maintaining its performance advantage in most cases. These findings underscore the importance of incorporating attention mechanisms in graph-based models for epidemic forecasting and highlight the potential of GAT for future research in this domain.

### 4.6 Discussion

The proposed GAST model offers several advantages over traditional influenza prediction models, which often focus on either spatial or temporal information in isolation. Even for models that consider the joint impact of spatiotemporal information, they often fail to effectively leverage the complex interactions between the two dimensions. In contrast, GAST leverages graph neural networks and dilated convolution modules to better capture and utilize spatiotemporal dependencies during the training process.

One of the key advantages of GAST is its global use of Graph Attention Networks (GAT) for information extraction and integration. By employing an attention mechanism, GAT dynamically determines the importance of each node’s neighbors, allowing the model to assign different weights when aggregating information from neighboring nodes. This enables GAST to capture more complex node relationships and better model the heterogeneous spatial dependencies in epidemic propagation. Moreover, GAST simultaneously considers propagation risk information, geographical relevance, and temporal dynamics through the GAT module, effectively exploring the interdependence between space and time to enhance influenza prediction accuracy.

Another significant advantage of GAST is the comprehensive evaluation of its predictive performance using diverse real-world datasets. We assessed the model’s accuracy and capability using three influenza datasets from different countries and a COVID-19 dataset from Spain. This diverse set of data sources allows for a thorough assessment and comparison of the model’s effectiveness, ensuring its predictive power across various conditions, regions, and socio-economic contexts. The results demonstrate the robustness and wide applicability of GAST in capturing the complex spatiotemporal patterns of epidemic spread.

However, it is important to acknowledge some limitations of the current study and identify potential areas for future improvement. One potential enhancement is the broader application of attention mechanisms throughout the model architecture. While GAST already incorporates GAT for information extraction and integration, the use of attention could be extended to other components, such as the spatial dependency modeling layer currently implemented with RNN. Replacing the RNN layer with Transformer or other attention-based models could enable parallel processing of the entire sequence while dynamically focusing on the most relevant elements, potentially leading to better capture of spatial dependencies and improved computational efficiency.

Another area for future exploration is the incorporation of dataset-specific pre-training. Although GAIT has demonstrated its ability to grasp general features across different datasets, it may benefit from further optimization for specific types of data. Pre-training the model on tasks specifically designed for each dataset could help capture the unique characteristics and distributions of the data more accurately. This targeted pre-training approach has the potential to further enhance the model’s predictive performance by leveraging dataset-specific knowledge.

In conclusion, the proposed GAST model represents a significant advancement in influenza prediction by effectively integrating spatial and temporal information through graph attention networks and dilated convolutions. The model’s superior performance, robustness, and wide applicability have been demonstrated through extensive experiments on diverse real-world datasets. While there is room for further enhancements, such as the broader application of attention mechanisms and dataset-specific pre-training, GAST offers a promising framework for accurate and reliable influenza forecasting. The insights gained from this study can inform public health decision-making and resource allocation in the face of epidemic outbreaks. As future work, we plan to explore the aforementioned improvements and extend the model’s application to other infectious diseases, ultimately contributing to more effective epidemic preparedness and response efforts.

## 5 Conclusion

In conclusion, this paper addresses the critical challenges in epidemic forecasting highlighted by the COVID-19 pandemic, specifically the need for models that can effectively integrate spatial and temporal data to provide accurate and actionable insights. Traditional epidemiological models, which rely mainly on time series analysis, have shown limitations in capturing the complex dynamics of disease spread. Our review of recent advancements underscores a shift towards integrating spatial information with advanced machine learning techniques, including graph neural networks and attention mechanisms, to enhance predictive accuracy.

The Graph Attention based Spatial-Temporal (GAST) model introduced in this study represents a significant advancement in epidemic forecasting. By harnessing the power of graph attention networks (GAT), GAST provides a comprehensive framework that captures the intricate interactions between spatial and temporal factors. This enables GAST to generate more detailed and accurate forecasts of epidemic spread compared to traditional models. Our extensive testing across multiple datasets demonstrates the model’s superior performance, particularly in short-term, daily forecasting of COVID-19 spread. This showcases the model’s potential to effectively inform public health interventions and policy implementations.

While the GAST model has demonstrated promising results, there are several avenues for future research that can further enhance its performance and applicability. One important direction is to incorporate additional data sources, such as mobility data, social media data, and demographic information, to capture a more comprehensive picture of the factors influencing disease spread. Another promising direction is to explore the application of the GAST model to other infectious diseases beyond influenza and COVID-19, adapting the model to capture disease-specific transmission dynamics and leveraging relevant data sources. Finally, there is an opportunity to explore the interpretability and explainability of the GAST model’s predictions, providing intuitive explanations to end-users and building trust among public health stakeholders. By addressing these challenges and opportunities, the GAST framework can be further refined and extended to become a powerful tool for informing public health decision-making in the face of epidemic threats.
